# Major Persistent 5′ Terminally Deleted Coxsackievirus B3 Populations in Human Endomyocardial Tissues

**DOI:** 10.3201/eid2208.160186

**Published:** 2016-08

**Authors:** Alexis Bouin, Yohan Nguyen, Michel Wehbe, Fanny Renois, Paul Fornes, Firouze Bani-Sadr, Damien Metz, Laurent Andreoletti

**Affiliations:** University of Reims Champagne-Ardenne, Reims, France (A. Bouin, Y. Nguyen, M. Wehbe, F. Renois, P. Fornes, F. Bani-Sadr, L. Andreoletti);; Centre Hospitalier Universitaire de Reims, France (Y. Nguyen, F. Renois, P. Fornes, F. Bani-Sadr, D. Metz, L. Andreoletti);; Centre Azm pour la Recherche en Biotechnologie et ses Applications, Tripoli, Lebanon (M. Wehbe)

**Keywords:** idiopathic dilated cardiomyopathy, coxsackievirus B3, 5′ terminally deleted populations, viruses, endomyocardial, heart, human, enterovirus

## Abstract

We performed deep sequencing analysis of the enterovirus 5′ noncoding region in cardiac biopsies from a patient with dilated cardiomyopathy. Results displayed a mix of deleted and full-length coxsackievirus B3, characterized by a low viral RNA load (8.10^2^ copies/μg of nucleic acids) and a low viral RNA positive-sense to RNA negative-sense ratio of 4.8.

Enteroviruses (EVs) are common human pathogens that are small, naked, single-stranded, positive-sense RNA viruses of ≈7,400 nt. Their genome is flanked on the 5′ end by a noncoding region (NCR), which is crucial for the initiation of the replication and translation of the virus genome (*1*). These viruses, especially group B coxsackieviruses (CV-B), are considered to be a common cause of acute myocarditis in children and young adults. This disease is a precursor to 10%–20% of chronic myocarditis cases and dilated cardiomyopathy (DCM), which is a leading cause of heart transplantation worldwide (*2*). The molecular mechanisms related to the switch from the acute to the persistent CV-B infection in human cardiac tissue are still unknown, but they could be related to terminal deletions on the 5′ NCR cloverleaf structure, resulting in low replication levels. One published report described the presence of EV-B strains containing genomic 5′ terminally deleted populations in heart tissues from a patient who had died of fulminant myocarditis (*3*). Several published studies reporting the presence of persistent EV infection in various human tissues, including heart tissue, did not investigate the presence of terminally deleted EV populations (*4*–*7*). We report a next-generation sequencing (NGS) strategy enabling a retrospective analysis of EV 5′ NCR of virus populations detected in heart tissue biopsies from a patient with idiopathic DCM (IDCM).

## The Study

In September 2011, a 47-year-old immune-competent woman was referred to University Hospital Center of Reims, Reims, France, because of stage 2 dyspnea (per New York Heart Association functional classifications, http://professional.heart.org/professional/General/UCM_423811_Classification-of-Functional-Capacity-and-Objective-Assessment.jsp) that began 1 month before. Transthoracic echocardiogram revealed a decreased left ventricle ejection fraction (LVEF) (45%) and a dilated left ventricle with a telediastolic diameter of 36 mm/m^2^. Coronary artery disease was ruled out by coronarography; no other etiologic causes of DCM were indicated by heart magnetic resonance imaging (*2*). Endomyocardial biopsies (n = 5) were performed at the time of hospitalization to assess the potential causes of IDCM (*8*). Classical and immunohistologic analyses indicated only a slight cardiomyocyte hypertrophy without myocarditis per the Dallas criteria (*8*). The clinical outcome was good, with a slight LVEF increase (54%) 4 years later after symptomatic treatment by β-blocker, therefore excluding the hypothesis of familial DCM.

Total nucleic acids were retrospectively extracted from flash-frozen cardiac tissues by using NucliSens easyMAG instrument protocols (bioMérieux, Marcy l’Etoile, France) according to the manufacturer’s instructions. Real-time quantitative reverse transcription PCR was performed for quantitative detection of total and strand-specific EV-genus genomic RNA according to previously published protocols (*7*). Generic EV-genus real-time quantitative reverse transcription PCR protocols showed the presence of EV total RNA detection with a low viral load of 8.10^2^/μg of total extracted nucleic acids and a low viral RNA positive-sense (RNA+) to RNA negative-sense (RNA–) ratio value of 4.8 (*7*).

Classical reverse transcription PCR sequencing of the viral protein 2 puff region enabled us to perform a genotypic identification of an original CV-B3 strain (GenBank accession no. KU608309); nucleotide and amino acid sequence homologies were 79% and 92%, respectively, with the CV-B3 Nancy strain (GenBank accession no. JX312064.1) (*3*,*9*). The genomic differences between the sample virus and the prototype strains ruled out the possibility of laboratory contamination events (*10*).

To identify the 5′ NCR terminal deletion, we performed a rapid amplification of cDNA ends (RACE) PCR followed by an in-house NGS strategy. RNA-backboned oligonucleotides (trP1 and trP1c) were linked on 5′ extremity of EV RNA+ and 3′ extremity of RNA–, respectively (Table). A retrotranscription step was then performed by using trP1 or AvcRev for a specific hybridization on viral RNA– or RNA+, respectively (Table). For the RACE PCR strategy, the amplification step was performed by using AvcRev and trP1 primers. For the NGS strategy, the amplicons were generated by using 2 successive PCRs (forward primers: A+AvcRevBC2–1 and A+AvcRevBC2–2 for the first and second PCRs, respectively; reverse primer: trP1) (Table; Technical Appendix). The libraries obtained were sequenced by using an Ion PGM sequencer (Life Technologies, Saint Aubin, France) according to the manufacturer’s instructions. Only reads containing both A and trP1 sequences were selected and aligned against the 5′ NCR regions of human EV genomes by using CLC Genomics Workbench software version 8.5.1 (CLC Bio, Aarhus, Denmark). 

**Table:** Primer sequences used to detect and identify 5′ terminal deletions in enterovirus populations detected in cardiac tissue of a patient with idiopathic dilated cardiomyopathy, Reims, France, September 2011

Primers	Oligonucleotide sequences
trP1 RNA*	5′H-CCTCTCTATGGGCAGTCGGTGAT-3′
trP1c RNA*	5′P-ATCACCGACTGCCCATAGAGAGG-3′H
trP1	5′-CCT CTC TAT GGG CAG TCG GTG AT-3′
AvcRev	5′-AACAGGCGCACAAAGCTACCG-3′
A+AvcRevBC2–1	5′-CGACTCAGTAAGGAGAACGATAACAGGCGCACAAAGCTACCG-3′
A+AvcRevBC2–2	5′-CCATCTCATCCCTGCGTGTCTCCGACTCAGTAAGGAGAACGATAAC-3′


*RNA backbone oligonucleotides.

Our results estimated a major proportion (84.8%) of reads presenting with a terminal 48-nt deletion associated with minor proportions of reads deleted of 15 nt (14.3%) and nondeleted (0.9%) (Figure). Because read lengths could induce variation in the amplification efficiency, our NGS strategy corresponded to a semi-quantitative detection of the EV populations. We concluded that the persistent viral population in the cardiac samples consisted of a major proportion of deleted populations, with deletions ranging in size from 15 to 48 nt (Figure).

**Figure:**
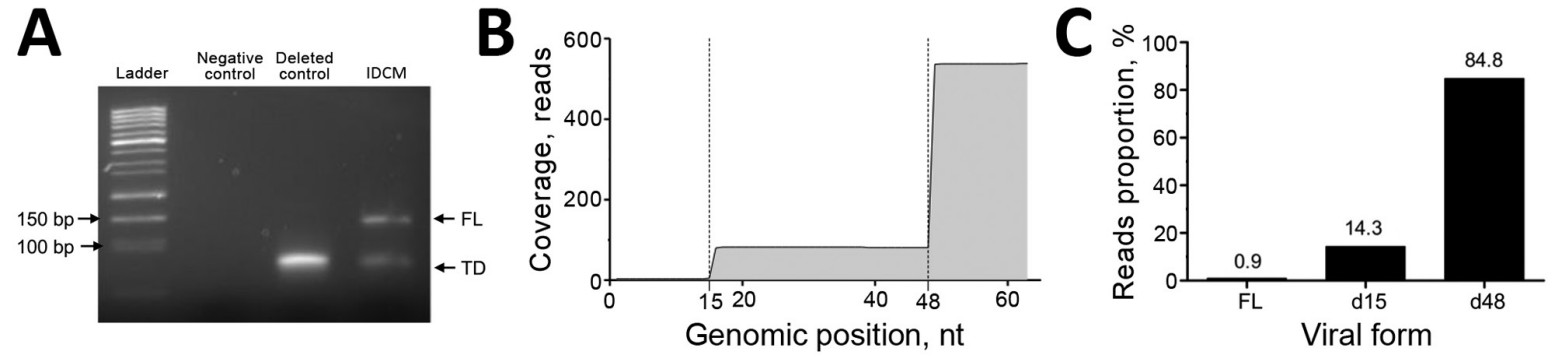
Identification of the major and minor 5′ terminally deleted or full-length enterovirus populations detected in the cardiac tissues of a patient with IDCM, Reims, France, September 2011. A) Gel electrophoresis analysis (4% agarose) of amplicons generated by using the rapid amplification of cDNA ends PCR strategy. Deleted control lane, synthetic RNA presenting with a 50-nt terminal deletion (*3*); IDCM lane, rapid amplification of cDNA ends PCR analysis of extracted RNA from heart tissues of the IDCM patient. B) Coverage data obtained by next-generation sequencing analysis of the cardiac tissue taken from IDCM patient. Reads obtained were filtered by bar code first to obtain 8,512,538 reads. A second discriminant step selected only reads containing both A and trP1 sequences, eliminating all artifacts resulting from early stops of the polymerase during amplification or sequencing. Following these steps, 7,354,283 reads were selected and aligned against the aligned human enterovirus group B genomes by using CLC Genomics software (CLC Bio, Aarhus, Denmark). Of these reads, only 538 were successfully aligned against enterovirus group B sequences and, more specifically, against coxsackieviruses B3 strain Nancy (Gen Bank accession no. JX312064.1). We observed that the persistent viral population in the cardiac samples consisted of a major proportion of deleted populations, with deletions ranging from 15 to 48 nt. C) Reads proportion obtained for each viral form from the IDCM patient. Coverage data were used to identify the full-length and deleted viral forms and indicate each populations’ proportions. IDCM, idiopathic dilated cardiomyopathy; FL, full-length; TD, terminally deleted.

## Conclusions

Our study demonstrated the existence of major 5′ terminally deleted CV-B3 populations in endomyocardial tissues taken from a patient with IDCM at the time of her clinical diagnosis. We observed low viral RNA load values associated with a low viral RNA+ to RNA– ratio (<5), indicating that these viral forms were compatible with persistent endomyocardial EV-B populations (*7*).

One previous study reported a detection of 5′ terminally deleted CV-B2 populations ranging in size from 22 to 36 nt in cardiac tissues taken from an immune competent patient in Japan who died of fulminant myocarditis (*3*). We demonstrated the existence of major persistent cardiac EV-B viral populations characterized by 15 and 48 nt deletions in the 5′ NCR at the time of chronic DCM (Figure). These deletions induce a loss of a part of the 5′ untranslated region cloverleaf structure, but the viral RNA polymerase (3CD) binding site remains intact (*11*). Such terminally deleted populations have been previously described in persistent murine cardiac infection characterized by low RNA+ to RNA– ratio associated with a low production level of infectious particles in cell cultures. Furthermore, murine and in vitro models have demonstrated that EV-2A proteinase is sufficient to induce DCM (*12*,*13*). As a result, low replicative terminally deleted viral forms could be generated and selected in heart tissues during the early acute viral replication phase (myocarditis) and could establish an ongoing persistent human cardiac infection leading to chronic myocarditis and the DCM clinical phase. Moreover, the NGS strategy showed that these 5′ terminally deleted populations were associated with a minor full-length virus population, which could potentially affect persistence by acting as a helper virus through genomic transcomplementation (*14*) or genomic recombination mechanisms (*15*). Persistent low replicative EV-B deleted and undeleted collaborative populations might contribute to the pathogenesis of unexplained DCM cases.

The clinical data associated with our molecular results argued for a CV-B3–induced DCM stage that developed several years after an undiagnosed clinical CV-B3–related myocarditis event. Taken together with the unique published CV-B2–induced myocarditis case, our findings support the hypothesis that the emergence and the selection of terminally deleted EV-B forms could occur during the early acute viral replication phase and might explain the pathophysiological progression from acute viral myocarditis to the DCM phase, in which cardiac persistent terminally deleted virus populations would be advantaged (*3*). Further NGS investigations on a large number of cardiac tissues of myocarditis and DCM adult patients might enable a better understanding of the molecular mechanisms implicated in cardiac EV persistence.

Technical Appendix Schematic representation of the next-generation sequencing library generation steps performed on total nucleic acid extracted from a flash-frozen cardiac tissue of a patient with idiopathic dilated cardiomyopathy, Reims, France, September 2011.
