# Supplementary material for: Major Persistent 5′ Terminally Deleted Coxsackievirus B3 Populations in Human Endomyocardial Tissues
Source: Emerg Infect Dis. 2016 Aug;22(8):1488–90. doi: 10.3201/eid2208.160186 (PMC4982168; doi:10.3201/eid2208.160186)
Supplement: Technical Appendix — Schematic representation of the next-generation sequencing library generation steps performed on total nucleic acid extracted from a flash-frozen cardiac tissue of a patient with idiopathic dilated cardiomyopathy, Reims, France, September 2011. [file 16-0186-Techapp-s1.pdf]

Article DOI: http://dx.doi.org/10.3201/eid2208.160186

Major Persistent 5′ Terminally Deleted Coxsackievirus B3 Populations in Human Endomyocardial Tissues

Technical Appendix

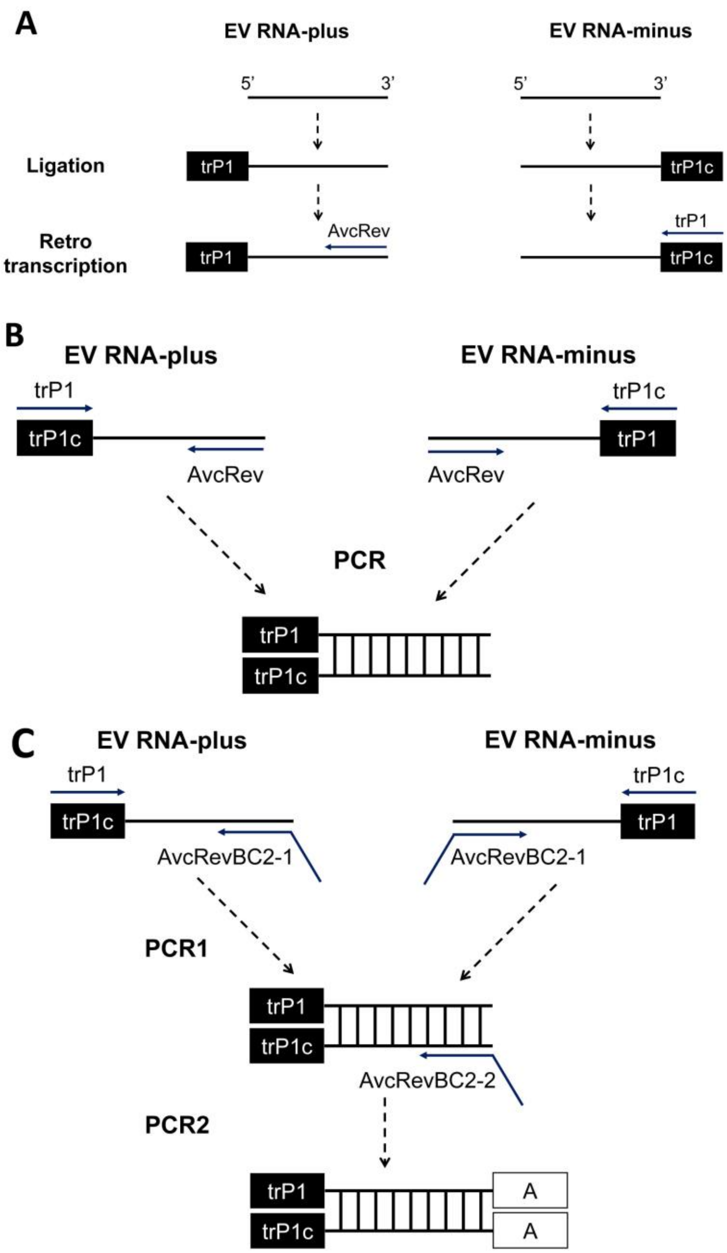

Technical Appendix Figure. Schematic representation of the next-generation sequencing library generation steps performed on total nucleic acid extracted from flash-frozen cardiac tissues of a patient with idiopathic dilated

cardiomyopathy, Reims, France, September 2011. A) Adapter ligation and cDNA generation: 2 different reactions were performed to amplify negative RNA and positive RNA. TrP1 adapter was ligated to the 5′ extremity of extracted positive RNAs, and trP1c (trP1 complementary sequence) was ligated to the 3′ extremity of negative RNA. A retro transcription step was performed by using different primers. AvcRev bound to the positive RNA and allowed the generation of a cDNA of the 5′ noncoding region until the terminal extremity (trP1 ligated sequence). For negative RNA, a primer consisting of trP1 bound to the ligated adapter and gives a cDNA of the complete 5′ noncoding region. B) Rapid amplification of cDNA ends PCR: a classical PCR using primers trP1 and AvcRev was performed on cDNA generated from positive and negative RNA ligation and enabled the amplification of the 5′ terminal genomic region. The length of the amplicons was assessed by 4% agarose gel electrophoresis (Figure, panel A). C) Next-generation sequencing library amplification: to be sequenced our amplicons needed to carry 1 different adapter on each terminal extremity (trP1 and A). The first adapter, trP1 was added during the ligation reaction. The second adapter (sequence A) was added in 2 PCR assays; the first reaction aimed to amplify efficiently the interest region, with the addition of the first part of the adapter, and the second reaction added the remaining part of the adapter. EV, enterovirus.
